# Estimation of the Solow-Cobb-Douglas economic growth model with a Kalman filter: An observability-based approach

**DOI:** 10.1016/j.heliyon.2019.e01959

**Published:** 2019-06-21

**Authors:** Rodrigo Munguía, Jessica Davalos, Sarquis Urzua

**Affiliations:** a Department of Computer Science, CUCEI, UdeG, 44430, Guadalajara, Mexico; b Centro Universitario de Ciencias Económico Administrativas, UdeG, 45100, Zapopan, Mexico; c Autonomous University of Guadalajara, Mexico

**Keywords:** Applied mathematics, Dynamical system, Systems theory, Economics, Economic growth

## Abstract

This work presents a novel approach for estimating the Solow-Cobb-Douglas economic growth model. In this case, an Extended Kalman Filter is used for estimating, at the same time, the time-varying parameters of the model and the system state, from subsets of partially available economic data measurements. Different from traditional econometric techniques, the proposed EKF approach is applied directly to a state-space representation of the original nonlinear model, where all the model parameters are treated as time-varying parameters. An extensive nonlinear observability analysis was carried out in order to investigate the different subsets of measurements that can be used for estimating the state of the system, and also, in order to find out theoretically necessary conditions to achieve the observability system property. Experiments with real macroeconomic data are presented in order to validate the proposed approach. While the observability analysis offer theoretically conditions for system observability, the experimental results suggest that between the subsets of available economic data, some specific economic data are more relevant than others for better estimating the model.

## 1 Introduction

One of the most important models of economic endogenous growth is the Solow-Swan model [1, 2]. The Solow-Swan model tries to explain the dynamics of long-run economic growth, as a result of investment capital, labor or population growth and the increment of productivity, also known as productivity factor or technological progress. The Solow-Swan model is considered to have attractive mathematical properties. It consists in a single nonlinear ordinary differential equation that models the evolution of the per capita stock of capital. Another economic growth models as the Ramsey-Cass-Koomans [3, 4] model can be viewed as extensions of the Solow-Swan model.

Parameter estimation plays a critical role in accurately describing system behavior by mean of mathematical dynamical models such as the Solow model. In the literature, different techniques have been used to carry out the parametric identification for the Solow growth model. Dwan and Gerdes [5] used the stochastic frontier production function to simultaneously estimate the technical efficiency together with the other production parameters. This for the case of the United States firms from 1970 to 1989. The authors found out a significant decline in the technology index for this period. On the other hand, Balistreri et al. [6] used time series with the objective of estimating in a consistent way a complete set of capital-labor substitution elasticities for the United States economy. Their calculations revealed the possibility of an aggregation bias, suggesting a reconsideration of averaging methods in flexible aggregation models. As well, Park and Ryu [7] used an approximation of meta-production function and maximum likelihood to separately identify the parameters related to production elasticities and those related to returns to scale. They found out evidence of increasing returns in the early stage of development of East Asian economies. In their work, Everaert and Nadal [8] sought to estimate the production function for the French business sector and the Total Productivity Factor (TFP) as a latent variable. To achieve this, the authors estimated the production function jointly with the TFP. The latter is specified as a latent variable through a self-progressive process of time series. On the other hand, Díaz et al. [9] observed the existence of convergence behavior at the local, regional or national level in the European Union by using Durbin's spatial multilevel estimation. The authors were able to identify a general behavior of convergence, however, several countries showed no evidence of significant internal convergence. While Deniz et al. [10] through a principal components analysis, concluded that the variables that capture the effects of human and physical capital generally produce a positive impact on the growth of 73 countries of the sample during the period of 1960–2014.

The Kalman filter is an algorithm developed by Rudolf E. Kalman [11], that is intended to estimate the state of a dynamic system (including the not measured hidden states), by mean of measurements observed over time, containing statistical noise and other inaccuracies. The Kalman filter has several applications in technology. Also, it is a mathematical tool widely applied in time series analysis and used in fields as signal processing and econometrics. In this latter case, the Kalman filter has been used in different ways in the economic area. Examples of this can be named, like the work of Yang and Den Chen [12]. They used it in a simultaneous demand-supply model. The method used shows how the exogenous variables affected the endogenous variables of price and quantity over time. In turn, Sanz [13] carried out the dynamization of the gravity equation with the Kalman filter to estimate the effects of the entry of Spain into the EEC produced on the manufacturing trade from 1986 to 1992. The proposed transformations of Box -Cox and Kalman filter were statistically appropriate for the Spanish case. Likewise, the results obtained from the impact of trade were more credible than with other approaches. Also, in his work, Wakashiro [14] estimated the price elasticity of electricity demand for each group in the manufacturing sector in Japan by using the partial adjustment model and the Kalman filter. The demand of this sector turned out to be more elastic than in the aggregate of industrial sectors. Of course, there are other interesting nonlinear filtering methods that have been used with success in several kinds of applications. The unscented Kalman filter (UKF) [15] uses a deterministic sampling technique, known as the unscented transformation, to choose a minimal set of sample points (called sigma points) around the mean. A new mean and covariance estimate is computed from the sigma points that are propagated through the non-linear functions. An example of the application of the UKF can be found in [16]. Another family of algorithms used to solve filtering and estimation problems are the Particle filters (PF) methods. Particle filtering uses a set of particles (also called samples) to represent the posterior distribution of some stochastic process given noisy and/or partial observations. The state-space model can be nonlinear and the initial state and noise distributions can take any form required. Examples of the application of the PF are: [17] and [18].

In this work, an Extended Kalman Filter (EKF) is used for estimating the system state of a Solow-Cobb-Douglas economic growth model. The proposed approach is intended to simultaneously estimate the time-varying model parameters, as well as the state of the dynamic system from a subset of available economic data measurements. First, a nonlinear state-space representation of the Solow-Cobb-Douglas model is derived. Different from traditional econometric methods (e.g. linear regression) where the system parameters are assumed to be constant over time, in the proposed state-space representation, all the system model parameters are defined as state variables in order to treat them as time-varying parameters. Based on the state-space representation, the observability properties of the system defined by the Solow-Cobb-Douglas economic growth model, are investigated by mean of an extensive nonlinear observability analysis. From this analysis, the necessary conditions for achieving the property of observability are derived, which in turn, is needed for estimating the full system state from different subsets of measured economic data. Finally, the system configurations that presented full observability are experimentally validated by applying the proposed EKF to real macroeconomic data.

This work presents different novelties. For instance, to our knowledge, this is perhaps the first time that it is proposed the application of a nonlinear filtering technique for estimating the Solow-Cobb-Douglas economic growth model and the first time that the observability properties of this nonlinear model are investigated.

In this work, an EKF has been chosen as estimation technique since, in our opinion, it represents a good starting point for investigating the application of the nonlinear filtering estimation methodology to this kind of problem. However, it is important to note that another nonlinear filtering estimation techniques could also be used for the same purpose. In particular, the theoretical findings obtained from the observability analysis can be useful not only for studying the applicability of the EKF but also for investigating the application of other state-space based estimation techniques (e.g. Particle Filtering, Unscented Kalman Filtering, etc.) to this class of economic models. The above, since the observability is an inherent property of the system and, therefore, it is independent of the estimation technique.

Also, an additional result suggests that the Cobb-Douglas production function can be estimated by using the methodology proposed in this work, as an alternative to traditional econometric methods (e.g. [19]) used for the same purpose.

The work is organized as follows: Section 2 presents the development of the proposed state-space representation of the nonlinear Solow-Cobb-Douglas economic growth model. Section 3 presents the nonlinear observability analysis of the Solow-Cobb-Douglas model. Section 4 summarizes the Extended Kalman Filter estimation technique. Section 5 presents the results of the experiments obtained with real macroeconomic data. Final remarks are presented in Section 6.

## 2 Model

A relevant equation of the Solow model is the equation of capital accumulation [20]:

(1) $$\dot{K}=\frac{\partial K}{\partial t}=I-\delta K$$

where *K* stands for the total capital over a period, *I* is the total investment on the capital formation over that period, and δ∈(0,1] is the depreciation rate of existing capital.

An important hypothesis of the model has to do with assuming that all the saving is invested on the capital formation. Therefore, S=I, where *S* is the saving, and:

(2) S=sY

where *Y* denotes the output of the economy, and s∈(0,1] is the saving rate. By substituting (2) in (1), the rate of change of the capital can be expressed in terms of the saving, as a ratio of the output of the economy:

(3) $$\dot{K}=sY-\delta K$$

A production function Y=F(.,.) relates quantities of physical output of a production process to quantities of production factors. In macroeconomics, aggregate production functions can be estimated for example to create a framework in which can be distinguished how much of economic growth can be attributed to the accumulation of physical capital and how much to attribute to advancing technology and labor.

In the Solow model it is assumed that a production function F(L,K), that depends on the labor *L* and the capital *K* presents constant returns to scale, meaning: F(λL,λK)=λF(L,K). In the Solow model, there are additional assumptions about the production function. These assumptions are important for the success of the model and even they are seen difficult to accomplish, they are employed in order to simplify the same.

Taking λ=L, and using the constant returns to scale assumption:

$$Y=F\left(L,K\right)=F\left(L\frac{K}{L},L\right)=LF\left(\frac{K}{L},1\right)$$

Then

(4) Y=Lf(k)

where $k=\frac{K}{L}$ denotes capital per capita, and $f\left(k\right)=F\left(\frac{K}{L},1\right)$. The common hypotheses about the function f(k) are that f(k) is an increasing function and strictly concave. Also, it is assumed that the Inada conditions are satisfied by f(k):

$$\underset{k\to \infty }{\text{lim}}f^{\prime }\left(k\right)=0\;\;\;\;\;\;\;\;\;\;\;\;\underset{k\to 0}{\text{lim}}f^{\prime }\left(k\right)=\infty$$

The fundamental equation of this economic growth model can be derived in the following manner:

$$\dot{k}=\frac{d}{dt}\frac{K}{L}=\frac{\dot{K}L-K\dot{L}}{L^{2}}=\frac{\dot{K}}{L}-\frac{K}{L}\frac{\dot{L}}{L}$$

Substituting (3) in the above expression, and considering *n* as the growth labor rate, $n=\frac{\dot{L}}{L}$, and $k=\frac{K}{L}$:

$$\dot{k}=\frac{sY-\delta K}{L}-nk$$

Substituting (4) in the above expression:

$$\dot{k}=\frac{sLf\left(k\right)-\delta K}{L}-nk=sf\left(k\right)-\delta \frac{K}{L}-nk=sf\left(k\right)-\left(\delta +n\right)k$$

Therefore, the rate of change of the capital stock by effective labor unity evolves according to the following differential ordinary equation:

(5) $$\dot{k}=sf\left(k\right)-\left(\delta +n\right)k$$

There are two equilibrium points $\dot{k}=0$, for the differential Eq. (5): k=0 and $k=k_{e}$. The last one must satisfy $sf\left(k_{e}\right)-\left(\delta +n\right)k_{e}=0$. The equilibrium point is unstable, while $k_{e}$ is stable.

One production functions F(K,L) that satisfies the requirements imposed by the Solow model is the production Cobb-Douglas function [21]:

(6) $$F\left(K,L\right)=AL^{\alpha }K^{\left(1-\alpha \right)}$$

where *A* is the total productivity factor, and *α* is the elasticity of work (labor).

Therefore, the final equation of the Solow-Cobb-Douglas economic growth model is obtained by including the Cobb-Douglas production function in 5. In this case, by considering that $f\left(k\right)=F\left(\frac{K}{L},1\right)=Ak^{\left(1-\alpha \right)}$, the following differential equation is obtained:

(7) $$\dot{k}=sAk^{\left(1-\alpha \right)}-\left(\delta +n\right)k$$

In order to apply the Solow-Cobb-Douglas economic growth model using actual economic data, commonly, the values of parameters *A* and *α* are identified from data *K* and *L*, by mean of the production function Y=F(K,L) using traditional econometric methods of parametric identification. For instance, the parameter *A* is calculated as the residual of the linear regression of production *Y*, as the dependent variable, depending on *K* and *L*. Additionally, *s*, *δ* and *n* are assumed to be known from economic data. In the above approach, by using linear regression, *A* and *α* are estimated as constant values. However, it is important to note that in reality, both parameters are varying over time.

In this work, we will treat all the parameters of the model 7 as varying parameters. In this case, for each parameter *p*, it will be assumed a dynamic $\dot{p}=v_{p}$, ${\dot{v}}_{p}=0$. That is, each parameter *p* has a constant rate of change $v_{p}$.

Considering the above assumption, the model defined in Eq. (7), can be expressed by means of the following state-space representation:

(8) $$\left\{ \begin{array}{l} \dot{k}=sAk^{\left(1-\alpha \right)}-\left(\delta +n\right)k\\ \begin{array}{cc} \dot{A}=v_{A} & {\dot{v}}_{A}=0\\ \dot{\alpha }=v_{\alpha } & {\dot{v}}_{\alpha }=0\\ \dot{s}=v_{s} & {\dot{v}}_{s}=0\\ \dot{\delta }=v_{\delta } & {\dot{v}}_{\delta }=0\\ \dot{n}=v_{n} & {\dot{v}}_{n}=0 \end{array} \end{array}\right.$$

Where $v_{A}$, $v_{\alpha }$, $v_{s}$, $v_{\delta }$, and $v_{n}$ are respectively the rate of change of the parameters: total productivity factor *A*, the elasticity of the work *α*, saving rate *s*, depreciation rate of existing capital *δ*, and growth labor rate *n*. It is important to note that the reason for assuming monotonic functions for modeling the dynamics of time-varying parameters is for keeping the state-space system model, defined in Eq. (8), simple enough but still allowing the parameters to be varying over time. Of course, in case of being available, specific dynamical models could be used instead, for representing the dynamical behavior of the time-varying parameters.

## 3 Analysis

In this section, the observability properties of the system defined by the Solow-Cobb-Douglas economic growth model will be studied by mean of a non-linear observability analysis. The objective is to find out the theoretical conditions needed for estimating this system from a set of measured economic data.

A system is defined as observable if the initial state $x_{0}$, at any initial time $t_{0}$, can be determined given the state transition model of the system $\dot{x}=f\left(x\right)$, the observation model of the system y=h(x), and the observations $z\left[t_{0},t\right]$, from time $t_{0}$ to a finite time *t*. In other words, if a system is fully observable then, all its internal states can be estimated in finite time from measuring the system output. Moreover, when a system is completely observable, the lower limit of the estimation error of a state will depend only on the noise of the system parameters and will not depend on the initial information about the state of the system.

The system state vector $x\in R^{11}$ to be estimated is:

(9) $${x=\left[\begin{array}{ccccccccccc} k & A & v_{A} & \alpha & v_{\alpha } & s & v_{s} & \delta & v_{\delta } & n & v_{n} \end{array}\right]}^{T}$$

with dynamics $\dot{x}=f\left(x\right)$ defined in (8). For the analysis, it will be assumed that the following measurement vector z can be available from macroeconomic data:

(10) $${z=[\begin{array}{ccccc} k & s & \delta & n & Y_{L} \end{array}]}^{T}$$

where k=K/L, and $Y_{L}=Y/L$. Note that in this case, in concordance to the standard approach, the parameters *A* and *α* will be treated as unknown parameters to be estimated from data.

Each component of the measurement vector z is predicted from the state vector x, by mean of the following measurement prediction models $h_{i}\left(x\right)$, where *i* is the index for the predicted component of z:

(11) $$h_{1}\left(x\right)=k\;\;h_{2}\left(x\right)=s\;\;h_{3}\left(x\right)=\delta \;\;h_{4}\left(x\right)=n\;\;h_{5}\left(x\right)=Ak^{\left(1-\alpha \right)}$$

In Hermann and Krener [22], it is demonstrated that a non-linear system is *locally weakly observable* if the observability rank condition rank(O)=dim(x) is verified, where O is the observability matrix.

The observability matrix O can be computed as:

(12) $$O={\left[\begin{array}{ccc} \frac{\partial \left(L_{f}^{0}h_{1}\right)}{\partial x}\frac{\partial \left(L_{f}^{1}h_{1}\right)}{\partial x}..\frac{\partial \left(L_{f}^{n}h_{1}\right)}{\partial x} & \frac{\partial \left(L_{f}^{0}h_{2}\right)}{\partial x}\frac{\partial \left(L_{f}^{1}h_{2}\right)}{\partial x}..\frac{\partial \left(L_{f}^{n}h_{2}\right)}{\partial x}\ldots & \frac{\partial \left(L_{f}^{0}h_{m}\right)}{\partial x}\frac{\partial \left(L_{f}^{1}h_{m}\right)}{\partial x}..\frac{\partial \left(L_{f}^{n}h_{m}\right)}{\partial x} \end{array}\right]}^{T}$$

where $L_{f}^{n}h$ is the *n-th*-order Lie Derivative [23], of the *m-th* scalar field $h_{m}$ with respect to the vector field f. It is important to note that, there is not a rule for determining the order of Lie Derivatives that should be used for composing the observability matrix. Therefore, the common approach is to add Lie Derivatives until the rank of the observability matrix remains constant. Also, it is very common that the complexity of the Lie Derivatives increases as its order increases. In this work, the observability matrices have been computed numerically by mean of the symbolic Matlab^®^ toolbox.

Table 1 shows all the system configurations from which a full rank matrix (rank(O)=dim(x)) has been found out. That means that for instance, the system state vector x can be observable by using only the subset of measurements $z_{g}=\left[\begin{array}{ccc} k & \delta & Y_{L} \end{array}\right]$ (See configuration **(g)** in Table 1). In this latter case, it should also be possible to treat *s* and *n* as unknown parameters that are estimated from the other available data.

**Table 1:** System configurations with full rank observability matrix for the system defined in 9 and 10.

Configuration	*k*	*s*	*δ*	*n*	$Y_{L}$
(a)	✓	✓	✓	✓	✓
(b)	✓	✓	✓	✓	✗
(c)	✓	✓	✓	✗	✓
(d)	✓	✓	✗	✓	✓
(e)	✓	✗	✓	✓	✓
(f)	✗	✓	✓	✓	✓
(g)	✓	✗	✓	✗	✓
(h)	✓	✗	✗	✓	✓

By analyzing Table 1, it can be observed two necessary conditions for achieving observability: i) It is necessary having at least measurements of the *δ* or *n* data, ii) In the absence of data *k* or $Y_{L}$, it is necessary having all the remaining type of observations.

A variation of the system dynamics defined in (8) has been also analyzed. In this case, the state variable w=δ+n is introduced. Therefore, the following state-space model $\dot{x}=f\left(x\right)$ is defined as:

(13) $$\left\{ \begin{array}{l} \dot{k}=sAk^{\left(1-\alpha \right)}-wk\\ \begin{array}{cc} \dot{A}=v_{A} & {\dot{v}}_{A}=0\\ \dot{\alpha }=v_{\alpha } & {\dot{v}}_{\alpha }=0\\ \dot{s}=v_{s} & {\dot{v}}_{s}=0\\ \dot{w}=w_{n} & {\dot{v}}_{w}=0 \end{array} \end{array}\right.$$

With a system state vector $x\in R^{9}$ to be estimated:

(14) $${x=[\begin{array}{ccccccccc} k & A & v_{A} & \alpha & v_{\alpha } & s & v_{s} & w & v_{w} \end{array}]}^{T}$$

In this case, the following measurement vector z is assumed to be available from macroeconomic data:

(15) $${z=\left[\begin{array}{cccc} k & s & w & Y_{L} \end{array}\right]}^{T}$$

and the following measurement prediction models $h_{i}\left(x\right)$ are defined:

(16) $$h_{1}\left(x\right)=k\;\;h_{2}\left(x\right)=s\;\;h_{3}\left(x\right)=w\;\;h_{4}\left(x\right)=Ak^{\left(1-\alpha \right)}$$

Table 2 shows all the configurations from which a full rank matrix (rank(O)=dim(x)) has been found for the system defined in (14) and (15). What is interesting to note about this analysis, is that introducing the variable *w*, the system becomes also observable by only using measurements from data *k* and $Y_{L}$ (See configuration **(n)**). That means that *δ* and *n* are indistinguishable from each other without the observation of one of these. On the other hand, it is very interesting to note that, aside from the above consideration, it should be possible to estimate the whole Solow-Cobb-Douglas model by using only the economic data commonly used for estimating the Cobb-Douglas production function Y=F(K,L). Namely: *Y*, *K*, and *L*. 4.5cm!

**Table 2:** System configurations with full rank observability matrix for the system defined in (14) and (15).

Configuration	*k*	*s*	*w*	$Y_{L}$
(i)	✓	✓	✓	✓
(j)	✓	✓	✓	✗
(k)	✓	✓	✗	✓
(l)	✓	✗	✓	✓
(m)	✗	✓	✓	✓
(n)	✓	✗	✗	✓

## 4 Methods

In Section 3, the observability properties of the Solow-Cobb-Douglas economic growth model were investigated. The observability is a measure of how well internal states of a system can be inferred from knowledge of its external outputs (measurements). In this case, several conditions of observability were found out from considering different sets of data measurements.

Based on the theoretical observability results previously presented, in this work, an Extended Kalman Filter (EKF) will be used for estimating the parameters of the Solow-Cobb-Douglas model from economic data. It is important to note, that the observability properties of a system are independent of the estimation technique used, and therefore, the theoretical results presented in this work should also be valid in order to be applied by using another estimation techniques.

In order to apply the EKF, and since the economic data is obtained in a discrete manner, a discrete-stochastic system model must be defined from the continuous dynamics $\dot{x}=f\left(x\right)$. In this work, the Euler method is used for this purpose, therefore:

(17) $$x_{k}=f\left(x_{k-1},n_{k-1}\right)=\left(x_{k-1}+f\left(x_{k-1}\right)\text{Δ}t\right)+n_{k}$$

The system measurement prediction model is defined by:

(18) $${h\left(x_{k},r_{k}\right)=[\begin{array}{cccc} h_{1}\left(x_{k}\right) & h_{2}\left(x_{k}\right) & \ldots & h_{n}\left(x_{k}\right) \end{array}]}^{T}+r_{k}$$

Let $n_{k}∼N\left(0,Q_{k}\right)$ and $r_{k}∼N\left(0,R_{k}\right)$ be the noise vectors that affect the state and the measurements, which are assumed to be mutually uncorrelated. Let Δt be the differential of time and *k* is the sample step.

The prediction stage of the EKF is defined by:

(19) $${\hat{x}}_{k}^{-}=f\left({\hat{x}}_{k-1},0\right)$$

(20) $$P_{k}^{-}=A_{k}P_{k-1}A_{k}^{T}+Q_{k-1}$$

The correction stage of the EKF is defined by:

(21) $${\hat{x}}_{k}={\hat{x}}_{k}^{-}+K_{k}\left(z_{k}-h\left({\hat{x}}_{k}^{-},0\right)\right)$$

(22) $$P_{k}=\left(I-K_{k}C_{k}\right)P_{k}^{-}$$

with

(23) $$K_{k}=P_{k}^{-}C_{k}^{T}{\left(C_{k}P_{k}^{-}C_{k}^{T}+R_{k}\right)}^{-1}$$

and

(24) $$\begin{array}{c} A_{k}=\frac{\partial f}{\partial x}\left({\hat{x}}_{k-1},0\right)\;\;C_{k}=\frac{\partial h}{\partial x}\left({\hat{x}}_{k}^{-},0\right) \end{array}$$

$z_{k}$ is the vector of actual measurements at step *k*, P is the covariance matrix of the system state and K is the Kalman gain.

## 5 Results

**Fig. 1:**
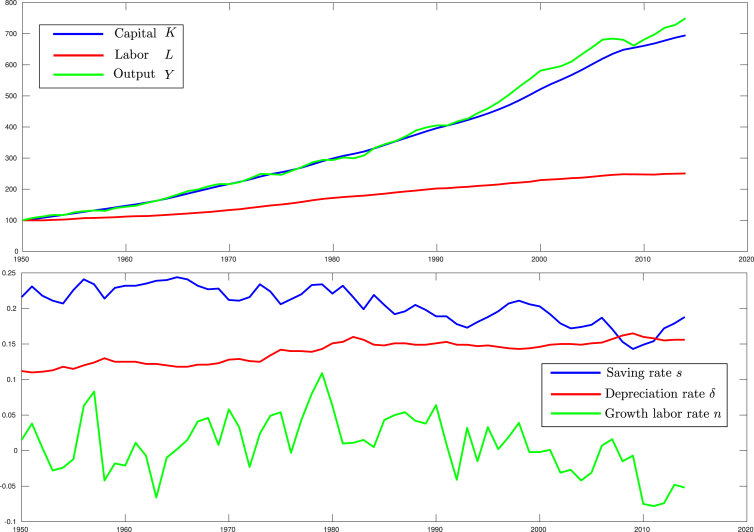
U.S. macroeconomic data from 1950 to 2015 for *K*, *Y*, *L*, *s*, *δ* and *n*.

In order to experimentally validate the study presented in this work, macroeconomic data obtained from [24] has been used. In particular, the annual times series from 1950 to 2015 of the U.S. national economy for the total capital *K*, the output of the economy *Y*, the labor *L*, the saving rate *s*, the depreciation rate of existing capital *δ*, and growth labor rate *n* are used as system measurements. Fig. 1 shows the former data. Note that for *K*, *Y*, *L*, indexed data is used. The time series of the productivity factor *A* was also obtained from the same database, but never it is used as input signal for the filter, but instead is used as an actual reference for evaluating the productivity factor estimated by the filter.

In order to perform the experiments, three periods of time have been defined:

• *Convergence period.* A convergence period from 1950 to 1980 is considered for the Extended Kalman Filter. In this case, the estimates produced by the filter, during this period, will not be considered for evaluating its performance but only for convergence purposes.
• *Testing period.* The data from 1980 to 2000 is used for evaluating the convergence properties of the filter. During this period, the estimates produced by the filter are compared with measured data. For instance, the productivity factor *A* estimated by the Filter, from the input signals, is compared with the actual productivity factor obtained from macroeconomic data.
• *Prediction period.* The data from 2000 to 2015 is used for evaluating the prediction properties of the filter. In this case, starting from the year 2000, the filter stop being “feeding” from any kind of measurements. Therefore, the estimates produced by the filter, during this period, are obtained solely by mean of its prediction stage (Eq. (19) and (20)). The estimates predicted by the filter are compared with the actual data available for this period.

**Fig. 2:**
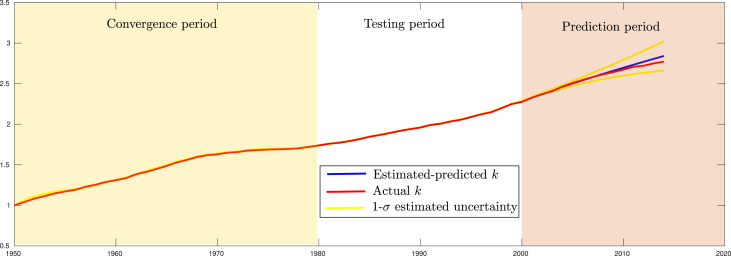
Comparison between the actual and the estimated value of *k*. During the prediction period, the filter is not updated with measurements.

Fig. 2 shows the evolution over-time of the state-variable *k* as is estimated and predicted by the filter, under its configuration **a**. Note that in this configuration the full set of measurements is available (see Table 1). In Fig. 2, the time series for the actual *k* (recall that) is also plotted. Additionally, the 1-*σ* uncertainty for *k*, obtained from the system covariance matrix P, is also plotted. The state variable uncertainty signal is useful for evaluating the consistency of the filter. Observe that from the year 2000, when the filter starts to work in prediction mode, the uncertainty starts to increase.

Table 3 summarizes the experimental results obtained for every system configuration described in Table 1. The *actual mean value* row shows the mean value computed directly from the available macroeconomic data (in the testing period) for each state variable. For instance, 1.442 is the mean value obtained from 1980 to 2000 for the productivity factor obtained from actual macroeconomic data. Below each actual mean value, the estimated value obtained from the filter is shown for each system configuration. For instance, observe that the system configuration **(h)** does not consider data measurements of the saving rate *s*, neither the depreciation rate of existing capital *δ* (See Table 1). In this case, these variables, as well as the productivity factor *A*, must be estimated by the filter from the other available data. In order to better highlight the observability properties of the filter, note that only the estimated mean values that are completely reconstructed from the measured data, are displayed for each system configuration.

**Table 3:** Prediction error of *k* and parameter estimated values, obtained for the system configurations (**a**-**h**).

Configuration	k∗	$\hat{k}$	$\hat{s}$	$\hat{\delta }$	$\hat{n}$	$\hat{A}$
*Actual mean value*		1.963	0.201	0.149	0.026	1.442
(a)	0.0086					1.453
(b)	0.0450					1.409
(c)	0.0091				0.046	1.528
(d)	0.0129			0.167		1.509
(e)	0.0151		0.208			1.455
(f)	0.5083	2.176				1.356
(g)	0.0130		0.164		0.005	1.452
(h)	0.0287		0.184	0.151		1.527

Also, in order to give an insight into the prediction performance of the filter, in Table 3 the column k∗ shows the mean squared error obtained for the estimated total capital *K* during the prediction period, from 2000 to 2015 (see Fig. 2). In this case, recall that during the prediction period the filter works solely based on the transition model $\dot{x}=f\left(x\right)$ and is not updated with any data measurements.

**Fig. 3:**
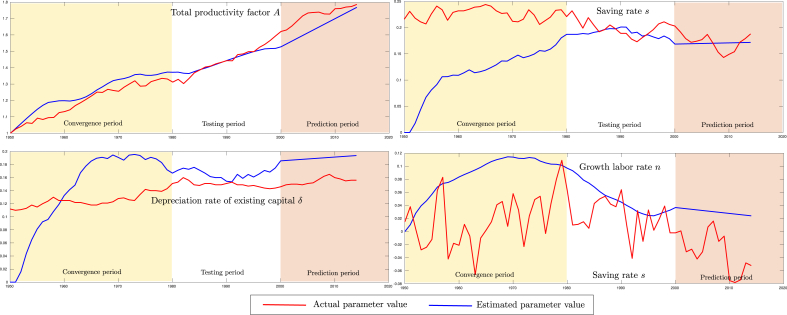
Comparison between the actual and the estimated parameters values over time.

Fig. 3 shows a comparison between actual and estimated values over time for the productivity factor *A*, the saving rate *s*, the depreciation rate of existing capital *δ*, and growth labor rate *n*, in cases where these state variables must be reconstructed by the filter. Note that for state variables *s*, *δ* and *n* the initial value is zero since there is no previous knowledge for them. On the other hand, by considering the Cobb-Douglas function $f\left(k\right)=Ak^{\left(1-\alpha \right)}$, and since indexed data is used for *K*, *Y*, and *L* (See Fig. 1), the initial value of *A* will be 1. Observe that the estimated values of the state variables do converge not very far from the actual value after a while.

Table 4 summarizes the experimental results obtained for every system configuration described in Table 2. In this case, recall that Table 2 summarizes the configurations obtained for the system defined in (14) and (15), in which the state variable w=δ+n is introduced. Other than that, Table 4 reads in the same way that Table 3.

**Table 4:** Prediction error of *k* and parameter estimated values, obtained for the system configurations (**i**-**n**).

Configuration	k∗	$\hat{k}$	$\hat{s}$	$\hat{w}$	$\hat{A}$
		1.963	0.201	0.175	1.442
(i)	0.0092				1.462
(j)	0.0271				1.401
(k)	0.0148			0.198	1.460
(l)	0.0162		0.196		1.489
(m)	0.3904	2.190			1.493
(n)	0.0113		0.002	-0.117	1.440

By analyzing the results displayed in Tables 3 and 4, it can be observed that the measurement of *k* appears to be, in practice, fundamental in order to estimate the remained state variables in a good manner (see the results of configurations **(f)** and **(m)** where *k* is not measured). It is important to recall that the observability property only tells us that the internal states can be estimated from measuring the system output in finite time, but no information is provided about the time of convergence of the estimates. For this reason, it is important to verify the theoretical results obtained from the observability analysis through the use of actual data. Also, as it can be expected, in general, it can be observed better estimation results when more variables are measured.

According to the theoretical results obtained from the observability analysis, by introducing w=δ+n, all the variables conforming the Solow-Cobb-Douglas model had to be estimated from the economic data commonly used for estimating the Cobb-Douglas production function. However, by observing the results of the configuration **(m)**, it can be seen that at least for this experiment, the variables *s* and *w* were not well estimated. On the other hand, the productivity factor *A* was very well estimated, and also the predicted *k* was close to its actual value. This result suggests that the Cobb-Douglas production function $f\left(k\right)=Ak^{\left(1-\alpha \right)}$ can be estimated using the methodology proposed in this work, as an alternative to the traditional econometric methods used for the same purpose.

In order to have a better insight into the above finding, the Cobb-Douglas function was estimated with the original Cobb-Douglas [21] data set. For comparison purposes, additional to the methodology proposed in this work, the parameters *A* and *α* where also estimated by means of a standard linear regression (See [19]), from the following linear model:

(25) log(Y)=log(A)+αlog(L)+(1−α)log(K)

Which can be derived by taking the logarithm of both sides of Eq. (6). With the linear regression the following values were obtained: *α* = 0.744606, *A* = 1.007.

**Fig. 4:**
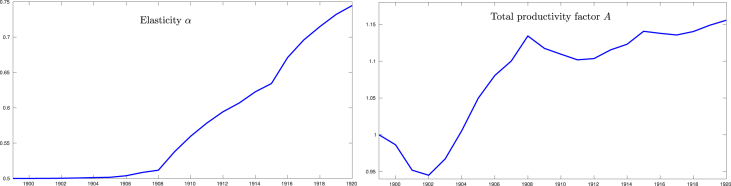
Estimation of the parameters of the Cobb-Douglas function with the original Cobb-Douglas data by mean of the proposed approach.

Fig. 4 shows the estimates obtained by means of the state-space model based methodology proposed in this work. In this case, despite the few data available (1899–1922), note that the value of the elasticity *α* converges from an initial value of .5 to a value near to the one obtained with linear regression. With the linear regression, the productivity factor *A* is calculated as the residual (i.e. a constant value) of the linear model. However, it is well known that the productivity factor commonly is increased over time. On the other hand, with the non-linear dynamic model proposed in this work, the productivity factor is modeled as an time-varying parameter. In this case, it can be noted that the estimates of *A* show an increment over time, which at least is consistent with that can be expected from the productivity factor of the U.S. economy in that period.

## 6 Conclusions

In this work, the application of the Kalman Filtering technique to estimate the Solow-Cobb-Douglas economic growth model from macroeconomic data has been investigated. Different from traditional econometric techniques where a linearized version of the original nonlinear model with constants parameters is used, the proposed EKF approach is applied directly to a state-space representation of the nonlinear model, where all the model parameters are treated as time-varying parameters. In this case, the proposed approach allow estimating the full system state, including the time-varying parameters, from different subsets of partially available economic data. Also, it allows capturing the dynamics of model parameters (e.g. productivity factor) that are commonly estimated as constant values, but in reality vary over time.

In order to investigate the different subsets of measurements that can be used for estimating the state of the system, an extensive nonlinear observability analysis was carried out for deriving necessary conditions to achieve the observability system property. In this case, if a system is fully observable then, all its internal states can be estimated in finite time from measuring the system output.

The proposed approach was tested with real macroeconomic data. While the observability analysis offer theoretically conditions for observability, the experimental results suggest that some economic data measurements are more relevant than others in order to better estimate the system state. Also, the theoretical and experimental results suggest that the Cobb-Douglas production function can be estimated by using the proposed approach, as an alternative to the traditional econometric methods used for the same purpose, with de advantage of capturing the time-varying behavior of the parameters. Based on the theoretical results that were presented, future work can consider the application of alternative state-space estimation techniques (e.g. particle filtering, Unscented Kalman Filtering, etc) for estimating Solow-Cobb-Douglas economic growth model.

## Declarations

### Author contribution statement

Rodrigo Munguia: Conceived and designed the experiments; Performed the experiments; Wrote the paper.

Jessica Davalos Aceves: Analyzed and interpreted the data; Wrote the paper.

Sarquis Urzua: Performed the experiments.

### Funding statement

This research did not receive any specific grant from funding agencies in the public, commercial, or not-for-profit sectors.

### Competing interest statement

The authors declare no conflict of interest.

### Additional information

No additional information is available for this paper.
